# A systematic review of the perforated duodenal diverticula: lessons learned from the last decade

**DOI:** 10.1007/s00423-021-02238-1

**Published:** 2021-06-23

**Authors:** Joshua R. Kapp, Philip C. Müller, Philippe Gertsch, Christoph Gubler, Pierre-Alain Clavien, Kuno Lehmann

**Affiliations:** 1 Department of Surgery and Transplantation, University Hospital of Zurich, Raemistrasse 100, CH-8091 Zurich, Switzerland; 2 Department of Gastroenterology, University Hospital of Zurich, Zurich, Switzerland

**Keywords:** Perforation, Duodenal diverticulum, Duodenum, Management

## Abstract

**Background:**

The perforated duodenal diverticulum remains a rare clinical entity, the optimal management of which has not been well established. Historically, primary surgery has been the preferred treatment modality. This was called into question during the last decade, with the successful application of non-operative therapy in selected patients. The aim of this systematic review is to identify cases of perforated duodenal diverticula published over the past decade and to assess any subsequent evolution in treatment.

**Methods:**

A systematic review of English and non-English articles reporting on perforated duodenal diverticula using MEDLINE (2008–2020) was performed. Only cases of perforated duodenal diverticula in adults (> 18 years) that reported on diagnosis and treatment were included.

**Results:**

Some 328 studies were identified, of which 31 articles met the inclusion criteria. These studies included a total of 47 patients with perforated duodenal diverticula. This series suggests a trend towards conservative management with 34% (16/47) of patients managed non-operatively. In 31% (5/16) patients initially managed conservatively, a step-up approach to surgical intervention was required.

**Conclusion:**

Conservative treatment of perforated duodenal diverticula appears to be an acceptable and safe treatment strategy in stable patients without signs of peritonitis under careful observation. For patients who fail to respond to conservative treatment, a step-up approach to percutaneous drainage or surgery can be applied. If surgery is required, competence in techniques ranging from simple diverticulectomy to Roux-en-Y gastric diversion or even Whipple’s procedure may be required depending on tissue friability and diverticular collar size.

## Introduction

The very first description of duodenal diverticula was published by French pathologist Auguste François Chomel in 1710. The duodenum is the second most common location for intestinal diverticula following the colon [1], and the prevalence of duodenal diverticula is estimated to be as high as 22% based on autopsy and ERCP series [2, 3]. The duodenal diverticulum can be classified according to congenital or acquired. Congenital diverticula, also known as true diverticula, result from prolapse of all layers of the bowel wall. These may be further subdivided into intra and extraluminal structures. Intraluminal diverticula are thought to arise due to aberrant formation of strictures or webs during duodenal anlage towards the end of the first month of human embryogenesis. The more common acquired diverticula result from prolapse of the mucosa and submucosa through the muscularis propria in areas of weakness thought to arise from perforating mesenteric vessels [4].

Complications of duodenal diverticula include ulceration, bleeding, perforation and inflammation with intestinal obstruction [5, 6]. Food stasis in periampullary diverticula can lead to cholangitis and pancreatitis [7, 8]. Some factors attributed to the low rate of inflammation of duodenal diverticula as compared to colonic diverticula include a higher rate of intraluminal duodenal flow, lower bacterial count and larger size of diverticula. Causes of duodenal diverticular perforation include diverticulitis, manipulation during endoscopy, ulceration, foreign bodies and back-pressure arising from distal bowel obstruction [9]. Perforation of duodenal diverticula is exceedingly rare with a sum total of 162 cases published in the entire world literature as of 2012. However, this entity has a high associated mortality estimated between 8 and 34% [9]. Consensus regarding optimal management is lacking and current management is guided by just a small series of cases, literature reviews and expert opinions. First to report on this potentially life-threatening complication were Juler et al., who in 1969 presented a series of 56 cases [10]. Duarte et al. contributed a further 45 cases in their 1992 review [11]. The last systematic review of this entity was published by Thorson et al. in 2012 [9]. Nearly a decade onwards, the present study takes a fresh look at the perforated duodenal diverticula by means of a systematic review of novel cases published since the last review in 2012. Here, we assess the evolution in its management and summarize the most recent examples of best practice.

## Methods

### Literature search strategy, study selection and data collection

**Fig. 1:**
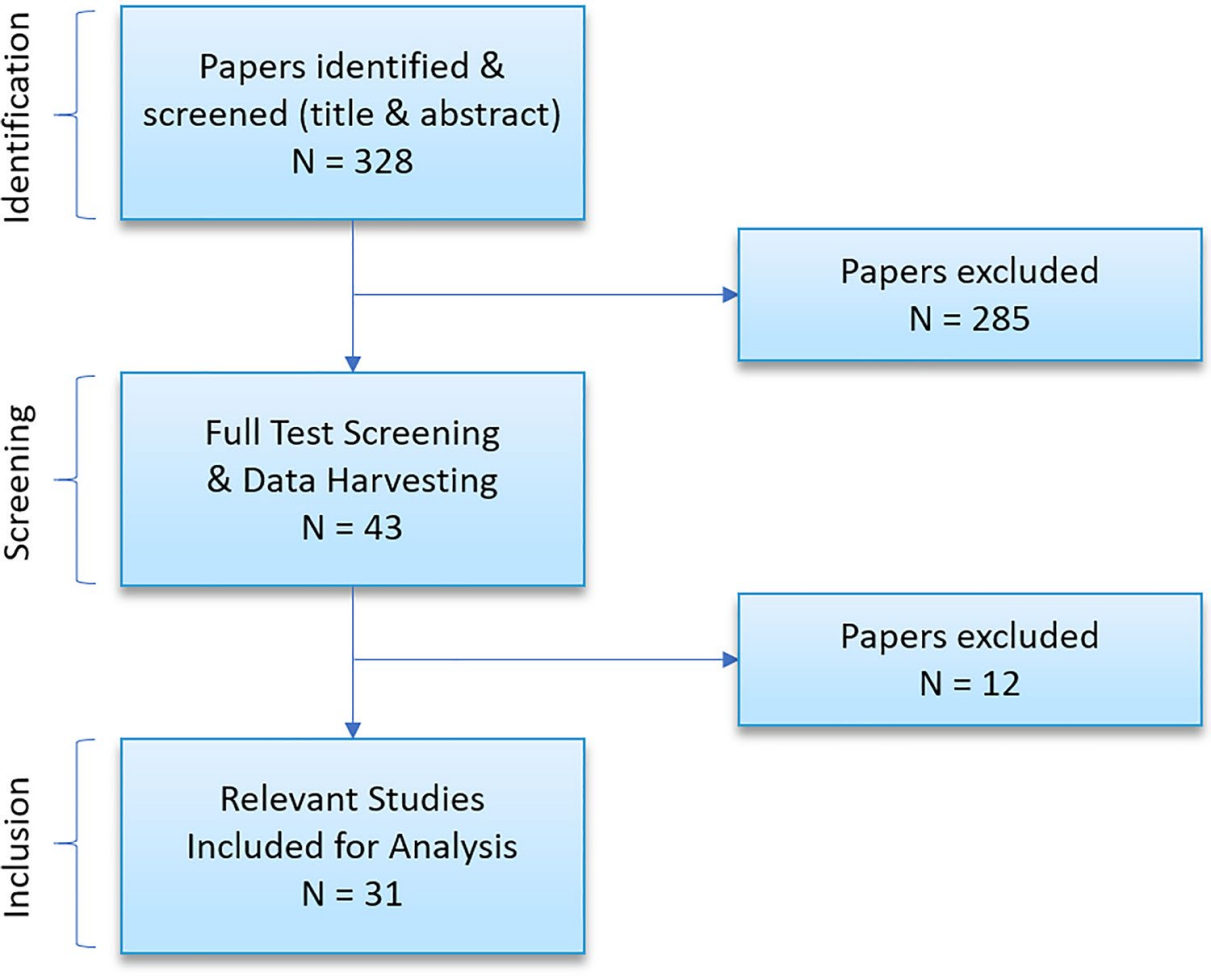
Search strategy. Among 328, 285 publications did not meet the inclusion criteria. The majority of these publications were excluded because they related to causes of duodenal perforation other than diverticula. Several articles were excluded because they focused on colonic diverticula. Other common reasons for exclusion were lack of appropriate data regarding treatment and outcomes, as well as publications in languages other than English, German or French. Of the 43 articles proceeding to full text screening, 12 were excluded due to inadequate details regarding therapy and outcome

A comprehensive and systematic search of the electronic databases MEDLINE (via PubMed) and Web of Science databases was carried out to identify relevant studies published during the last decade, between 2008 and 28th May 2020. The following inclusion criteria were applied: cases of adult (> 18 years) perforated duodenal diverticula; with information pertaining to treatment and clinical course; published in English, German or French. The search terms depicted in Fig. 1 were employed. References of included studies were analysed for additional relevant publications not identified in the original search. Eligibility assessment and data extraction was performed independently in an unblinded standardized manner by two reviewers. To avoid errors in data extraction, a double data-entry method was applied. Two authors compared the data and discussed discrepancies to achieve consensus. Articles already reported in previous systematic reviews, as well as those lacking sufficient information on diagnosis, management and outcome were excluded from the study. Information from each case was extracted including (1) year of publication, (2) presenting symptoms and signs, (3) diagnostic method, (4) aetiology, (5) size and location of perforation and treatment and (6) follow-up and complications. The anatomy of the duodenum (D1-4) was defined as follows: first part (D1) from pylorus to superior duodenal flexure, second part (D2) from superior to inferior duodenal flexure (including major and minor duodenal papilla), third part (D3) from inferior duodenal flexure to its crossing of the vertebral column and the subsequent fourth part (D4) to the duodenojejunal flexure. The primary outcome measure was the proportion of patients receiving operative versus non-operative treatment. Data compilation was performed using SPSS, version 25. Data reporting was conducted according to the PRISMA statement for reporting systematic reviews [12].

## Results

Data extraction from electronic databases yielded a total of 328 potential articles. Of these, 285 articles were discarded after reviewing the abstracts, and the full text of the remaining 43 citations were subsequently examined. Of these 43 articles, it appeared that 12 studies did not contain adequate information regarding patient diagnosis, management or outcome and thus did not meet the inclusion criteria. A total of 31 relevant citations involving 47 cases were included for final analysis (Table 1).

**Table 1:** Overall, 47 patients were included from published studies since 2008. This table details key information regarding the patient characteristics and treatment

Patient	Publication	Age/Gender	Peritonitis	1° Diagnostic	RP Fluid	RP Air	Aetiology	Location	Perforation	Management	Complications	LOS
1	Bahamonde et al. 2009 [28]	72F	No	CT	Y	Y	-	D2/D3	RP	CM	None	14
2	Barillaro et al. 2013 [29]	83F	No	CT	Y	Y	-	D3	RP	CM, step-up to lavage and drainage	None	30
3	Branco et al. 2017 [30]	80F	No	CT	Y	Y	-	D2/D3	Free	Diverticulectomy	None	7
4	de Perrot et al. 2012 [31]	92F	-	CT	Y	Y	-		RP	Diverticulectomy	None	-
5	de Perrot et al. 2012	86F	-	CT	Y	Y	-	D2	RP	Diverticulectomy	None	-
6	de Perrot et al. 2012	48F	-	CT	Y	Y	-	D2	RP	Diverticulectomy	None	-
7	de Perrot et al. 2012	75F	-	CT	Y	Y	-	D2	Free	CM	None	-
8	de Perrot et al. 2012	77F	-	CT	Y	Y	-	D2	RP	Diverticulectomy	None	-
9	de Perrot et al. 2012	69 M	-	CT	Y	Y	-	D2	RP	Diverticulectomy	Death	-
10	de Perrot et al. 2012	71F	-	CT	Y	Y	-	D4	RP	CM	None	-
11	Degheili et al. 2017 [32]	81 M	Yes	CT	N	Y	-	D2	RP	CM	None	14
12	Degheili et al. 2017	53F	-	CT	Y	Y	Iatrogenic	-	RP	Surgical drainage	None	40
13	Favre-Rizzo et al. 2013 [8]	82F	No	CT	Y	Y	Foreign Body	D3	RP	Diverticulectomy	None	14
14	Fujisaki et al. 2014[22]	69 M	-	CT	N	Y	-	D2	Free	CM, step up to R-Y-Duodenojejunostomy	Duodenal fistula	40
15	Glener et al. 2016 [33]	65F	Yes	CT	N	Y	-	D2/D3	RP	Diverticulectomy, Gastrojejunostomy	None	10
16	Gottschalk et al. 2010 [34]	81F	-	EGD	N	N	-	D2	Free	CM, step up to Diverticulectomy	None	-
17	Gulmez et al. 2016 [35]	22 M	Yes	CT	N	Y	-	D2/D3	RP	Diverticulectomy	None	-
18	Haboubi et al. 2014 [3]	78F	-	CT	N	Y	-	D2	Free	Diverticulectomy	Anastomotic leak	90
19	Kabelitz et al. 2020 [36]	47F	Yes	CT	Y	Y	Biliary stone	D2	RP	Suture repair, T-drain	None	12
20	Khan et al. 2018 [37]	82F	-	CT	Y	Y	-	D3	RP	CM, step up to diverticulectomy, duodenojejunostomy	Death	12
21	Kim et al. 2018 [38]	68 M	No	CT	Y	Y	-	D2/D3	RP	CM	None	17
22	Koh et al. 2016 [39]	81F		CT	N	Y	Bezoar	D2	RP	Omental patch, surgical drain	None	20
23	Majerus et al. 2016 [25]	65F	Yes	CT	Y	Y	Trauma	D2	RP	Diverticulectomy	None	12
24	Maki et al. 2020 [40]	94F	Yes	CT	N	Y	-	D2	RP	Diverticulectomy	None	24
25	Ming et al. 2012	77 M	-	CT	N	Y	-	D3/D4	RP	Duodenectomy	None	-
26	Moysidis et al. 2020 [41]	51F	No	CT	N	Y	-	D2	RP	Diverticulectomy	None	10
27	Moysidis et al. 2020	58F	-	CT	N	Y	-	D2	RP	CM	Pneumonia	26
28	Nepal P, et al. 2017	81F	Yes	CT	N	Y	-	D3	RP	Duodenectomy	Duodenal fistula	-
29	Perdikakis et al. 2011	58 M	-	MRI	N	Y	-	D2	NR	CM	-	-
30	Philip et al. 2019	70F	Yes	CT	Y	Y	-	D2	Free	Pancreaticoduodenectomy	None	10
31	Rossetti et al. 2013 [6]	91F	-	Surgery	-	-	-	D2	NR	Diverticulectomy	None	26
32	Rossetti et al. 2013	68F	-	Surgery	-	-	-	D2	NR	Surgical drain	Death	1
33	Rossetti et al. 2013	83F	-	CT	-	-	-	D2	NR	Diverticulectomy	-	18
34	Rossetti et al. 2013	78F	-	Surgery	-	-	-	D2	NR	Diverticulectomy	Leak	30
35	Rossetti et al. 2013	76F	-	CT	-	-	-	D2	NR	CM	-	16
36	Rossetti et al. 2013	65 M	-	Surgery	-	-	-	D2	NR	Diverticulectomy	-	15
37	Rossetti et al. 2013	48 M	-	CT	-	-	-	D3	NR	Diverticulectomy	-	22
38	Sahned et al. 2019 [42]	77F	No	CT	Y	Y	-	D2	RP	CM, step up to diverticulectomy	None	4
39	Sasaki et al. 2015 [43]	58 M	-	CT	N	Y	-	D3	RP	CM	-	-
40	Shirobe et al. 2017 [44]	52F	No	CT	Y	Y	Bilirubin Calculus	D2	RP	CM, drain	None	22
41	Costa et al. 2014 [21]	79F	No	CT	N	Y	-	D4	RP	Partial duodenectomy with duodenojejunostomy	None	12
42	Song et al. 2015 [45]	53 M	Yes	CT	Y	Y	Foreign Body	D2	RP	Diverticulectomy	None	12
43	Song et al. 2015	73 M	Yes	CT	N	Y	-	D2	RP	CM	None	22
44	Sugimoto et al. 2020 [46]	80F	Yes	CT	N	Y	Foreign Body	D2	RP	Lavage, drain	Urinary infection	35
45	Yang et al. 2015 [47]	69F	Yes	CT	N	Y	ERCP	D2	Free	Surgical drain	None	-
46	Yagi et al. 2019 [24]	66 M	No	CT	Y	Y	-	D2	RP	Pancreaticoduodenectomy	None	23
47	Yagi et al. 2019	52 M	Yes	Surgery	-	-	-	D2	RP	Suture repair, drain	Abscess, perforation of second DD	53

*CM* conservative management; *CT* computer tomography; *DD* duodenal diverticulum; *EGD* esophagogastroduodenoscopy; *ERCP* endoscopic retrograde cholangiopancreatography; *F* female; *M* male; *LOS* length of stay; *MRI* magnetic resonance imaging; *NR* not reported; *RP* retroperitoneal

### Baseline characteristics

**Fig. 2:**
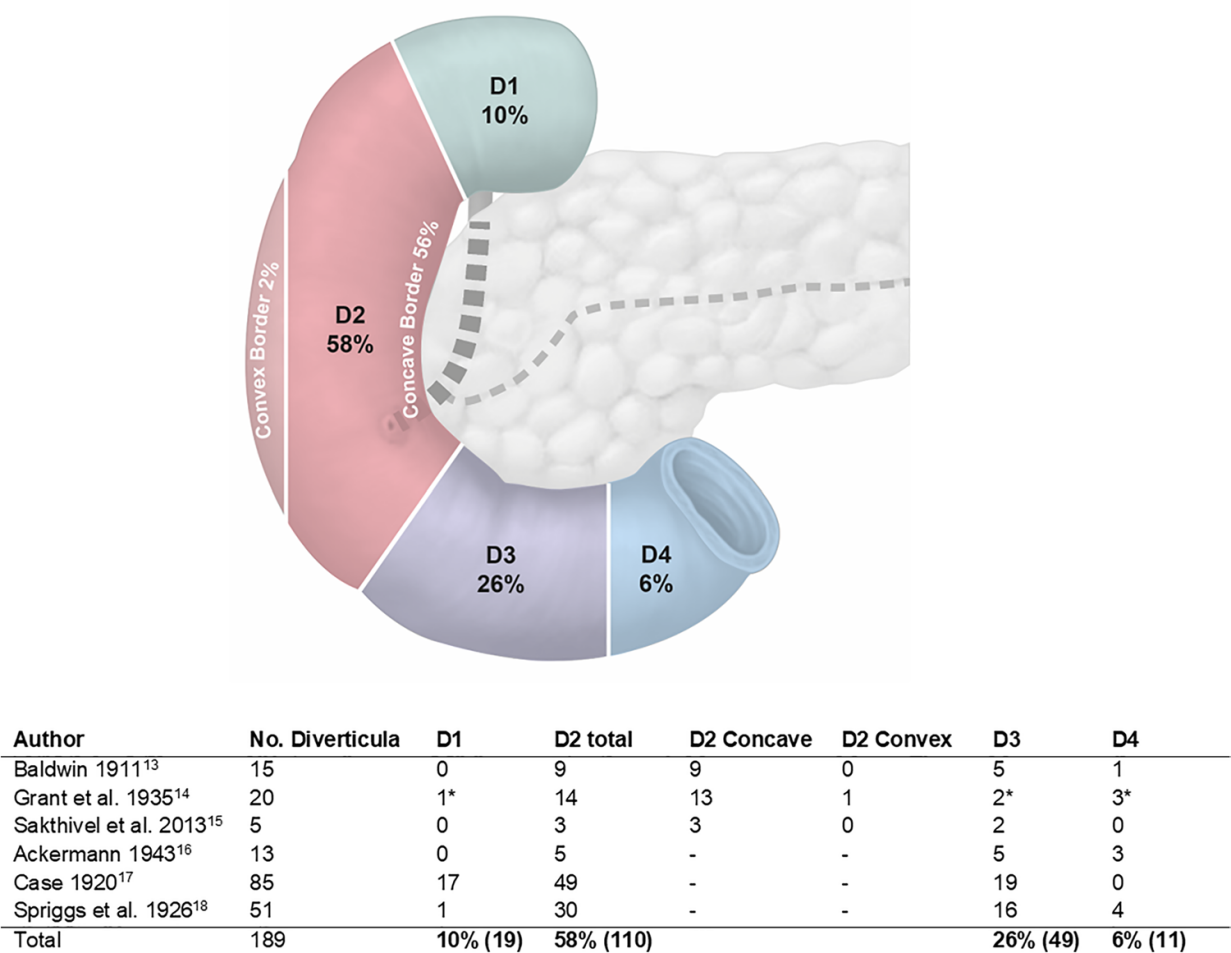
Anatomical distribution of duodenal diverticula. The majority of duodenal diverticula grow from the concave, pancreatic border of the duodenum, morphologically the mesenteric duodenal border. * Junction D1/D2; Junction D2/D3; Junction D3/D4 as reported by published autopsy series.—No further specific information available

**Fig. 3:**
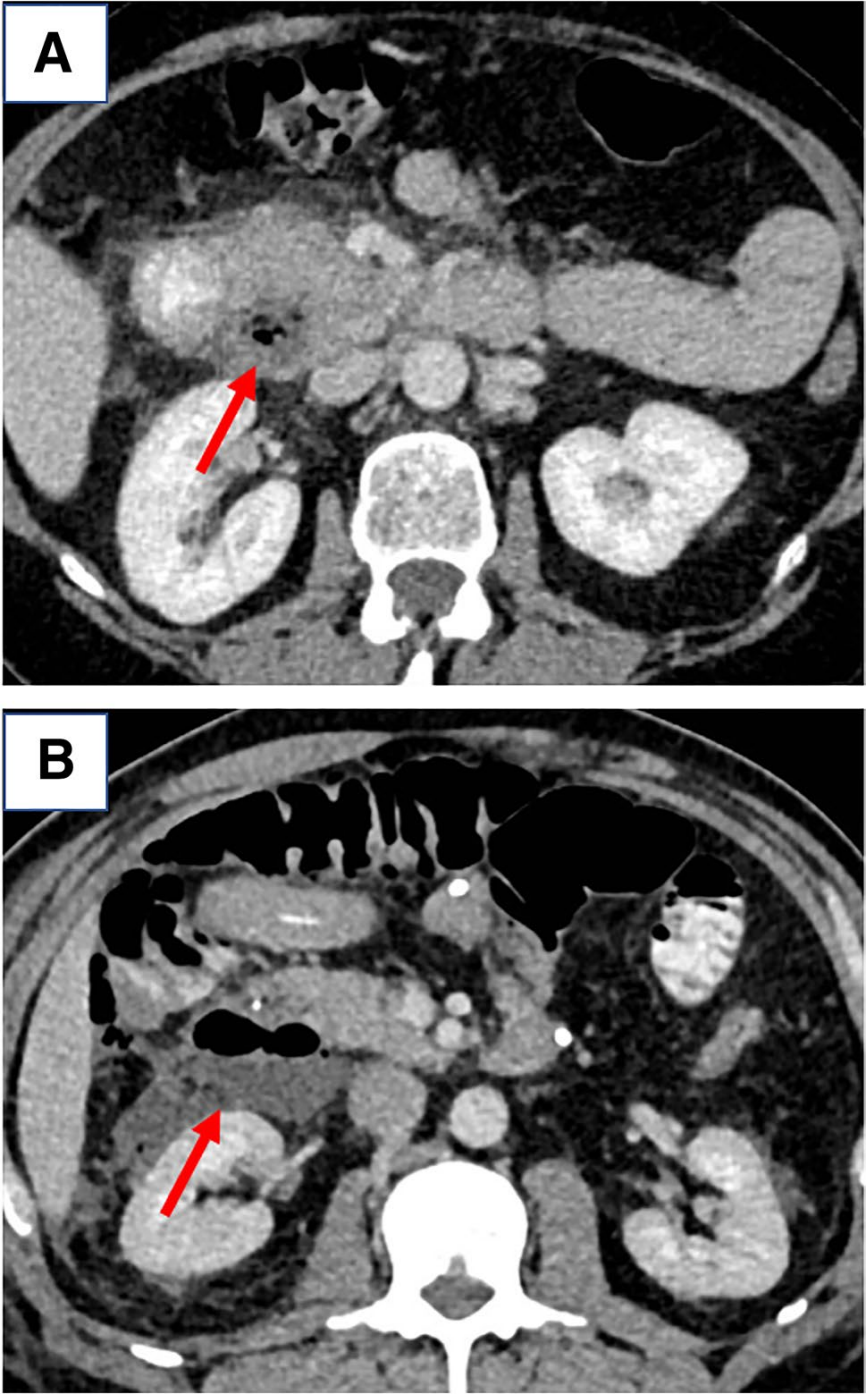
Selection of patients for initial conservative management. Imaging features of two patients successfully managed conservatively. **A** A 59-year-old patient was admitted with 24 h of vomiting and acute epigastric pain radiating to the back. Extraluminal, retroperitoneal air was found (arrow). Conservative therapy with bowel rest, jejunal feeding tube, intravenous broad-spectrum antibiotic- and PPI-therapy was established. **B** A 58-year-old female presented a brief history of epigastric pain. Clinical examination revealed a tenderness in the right upper quadrant. Again, extraluminal, retroperitoneal air was found on CT scan (arrow). The patient was managed with the same conservative regimen. She was discharged after 1 week and is asymptomatic at 8 months follow-up.

There is a known predilection for duodenal diverticula in older age. The average age in the present series was 70 (range 22–94) years (Table 1). Although an equal predilection for the sexes has been reported in the literature, the present series demonstrated a preponderance for female patients (70% vs. 30%). The majority (43/47) presented with acute abdominal pain and diverticula were located predominantly in the second part of the duodenum (Figs. 2 and 3) [13–18]. Computer tomography (CT) was the primary diagnostic method in 92% (43/47) of cases. In 65% (28/43) of said cases, CT enabled prospective diagnosis of the perforated diverticulum prior to intervention. Where reported, 97% of cases (36/37) showed free retroperitoneal air and 53% reported retroperitoneal fluid (20/38). In the 35% (15/43) cases where CT did not provide prospective confirmation, surgical intervention was required for diagnostic confirmation in all but a single case (**#**38) where esophagogastroduodenoscopy (EGD) was performed enabling prospective confirmation of the diagnosis. In the remaining 8% (4/47 cases), 1 case underwent emergency, exploratory laparotomy, 1 case did not report the information, and 2 cases (**#**16, **#**28) reported EGD as the primary diagnostic.

Precise information regarding the diameter of perforated diverticula was reported in 11 of 47 cases and was quantitated either by CT or morphological diagnosis. The reported diameters of perforated diverticula ranged from 2.8 to 76 mm (median 37 mm). Information regarding peritonitis was available for 23/47 cases, 13 presented with peritonitis and 10 without. Post-interventional imaging was reported in 32% (15/47), and follow-up endoscopy was reported in 9% (4/47) of the cases.

### Conservative management (CM)

Of the 47 cases in this series, 16 (34%) were either treated conservatively for the duration (11/47) or initially treated conservatively with subsequent step-up to surgery (5/47).

Only one patient with peritonitis was initiated and successfully treated on conservative management due to old age and presence of co-morbidities. Absence of peritonitis was specified in 9 of the other 15 patients, five of whom were successfully managed conservatively, four required step-up to surgery.

Of these conservatively treated cases, 9 were successfully managed with bowel rest and intravenous antibiotics, and 2 were successfully managed with adjunct endoscopic therapy (endoscopic repair of perforation and endoscopic stenting, respectively). The remaining 5 patients failed conservative therapy and required surgical intervention (Table 2). Conservative management using endoscopy was successfully applied in two cases (**#**39, **#**40) and unsuccessfully in one case (**#**16). Case 39 reported successful application of a novel approach involving endoscopic tissue shielding applied to diverticulum using polyglycolic acid sheet and fibrin glue. Case 40 reported successful application of endoscopic stenting of the common bile duct to protect against risk of impingement from an adjacent retroperitoneal abscess (V-system stent, Olympus Medical Systems, Tokyo). This was followed by stenting of the retroperitoneal abscess through the perforated diverticulum to ensure patency for drainage (Advanix double pigtail stent 18,207,106-7Fr × 7 cm, Boston Scientific, MA). The patient made a full recovery and stents were removed in the outpatient clinic on day 40 from admission.

**Table 2:** Parameters for decision to step up

Case #	Peritonitis at presentation	Time to step-up	Clinical parameter	Diagnostic parameter	Details
2	No	72 h	Onset of sepsis	Repeat CT	Onset of fever, leukocytosis, increase in abdominal pain after 72 h prompted repeat CT which strongly suggested locally confined perforation, prompting decision for surgery
14	NR	48 h	Persistent pyrexia	Repeat CT	Persistent pyrexia triggered repeat CT demonstrating free intraperitoneal and retroperitoneal gas prompting a diagnosis of perforation and decision for laparotomy
16	No	72 h	Acute Abdomen	CT	Onset of an acute abdomen 24 h after the last endoscopy prompting CT which demonstrated free intra-abdominal and retro-peritoneal air
20	No	NR	Haemodynamic instability	Repeat CT	Developed abdominal distention and became haemodynamically instable, prompting another CT which revealed retroperitoneal free fluid in region of duodenum thus providing the indication for surgery
38	No	72 h	Persistent abdominal pain	Repeat CT	The diagnosis was made via an upper endoscopy that showed a large periampullary duodenal diverticulum with purulent drainage. Due to persistent epigastric pain and tenderness with an interval increase in the retroperitoneal collection which was determined to be not amenable to percutaneous drainage

*CT* computer tomography; *NR* not reported

**Fig. 4:**
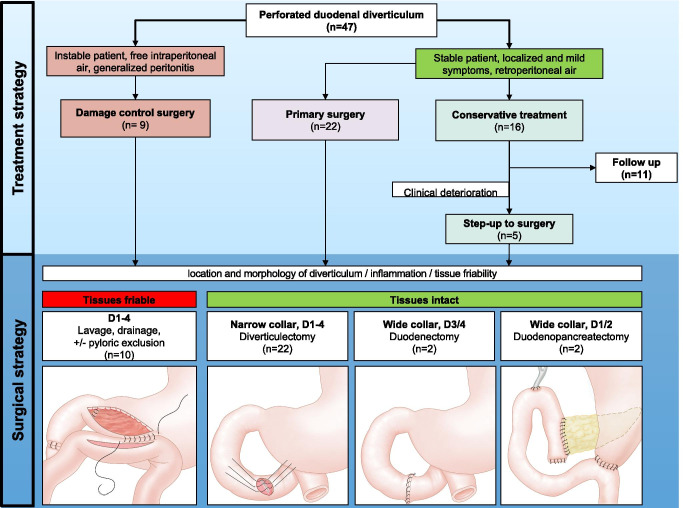
Patient stratification algorithm enabling a step-up approach. The algorithm differs between patients who are clinically stable without generalized peritonitis, who may be considered for conservative treatment, and potentially delayed elective surgical treatment. Absence of peritonitis, old age and presence of significant comorbidities were key reasons underpinning the decision for conservative management. The various technical options should highlight the complexity of the procedure, depending not only on the anatomical location (e.g. proximity to biliopancreatic duct) or morphology (width of diverticular collar) of the duodenal diverticula but also on the degree of tissue vulnerability at the time of exploration. * conservative treatment was defined as: intravenous antibiotic treatment, jejunal feeding tube or TPN, ± percutaneous abscess drainage. + there is no actual definition of a narrow or wide collar. However, a defect who, after surgical closure, will not narrow the lumen of the duodenum might be considered as a narrow collar

Absence of peritonitis, old age and presence of significant comorbidities were cited as the key reasons underpinning the decision for initial conservative management (Fig. 4). Factors influencing the decision to step-up to surgical therapy are detailed in Table 2.

### Surgical management (SM)

In total, 31/47 patients (66%) underwent SM from the beginning, and 5/47 (10%) patients underwent a surgical step-up after CM (Fig. 4). The various surgical techniques employed varied considerably. Diverticulectomy, with stapler, single- or double-layer transverse closure, was the most common surgical intervention (20/36), followed by lavage and drain placement (6/36). Additional measures to reduce gastric outflow were taken in all cases by means of nasogastric tube placement. More extensive surgery was only performed in 6 cases. This ranged from duodenectomy with duodenojejunostomy for cases involving D3/4 diverticula [19–21] to more complex gastric diversion by means of Roux-en-y duodenojejunostomy [22] and pancreaticoduodenectomy in two cases of complex perforated D2 diverticula [23, 24]. Extensive inflammation and tissue friability were the primary reasons attributed to performing more extensive resection over simple diverticulectomy in these cases. Two of these 6 cases were complicated by development of a duodenal fistula. In both cases the fistula resolved without further surgical intervention. Although information on tissue quality is only reported in a small minority (8/47) of the cases, tissue quality was reported as friable in 7/8 cases. Of the friable cases, 6/7 were confined to retroperitoneum, and 4/7 were reported to involve D2. Surgical management of these cases required lavage and drainage (case 2), duodenojejunostomy (cases 14,15, 41), gastrojejunostomy (case 28), foreign body removal via intraoperative endoscopy (case 44) and pancreaticoduodenectomy (case 46), respectively.

### Morbidity and mortality

Overall mortality, including both conservative and surgically managed patients (n = 47), was 6% (3/47). Mortality in patients receiving conservative treatment without escalation to surgery (n = 15) was 0%. When adjusting to include patients who were escalated to surgery, the associated mortality was 6% with just a single case (1/16) in which the 82-year-old patient died due to multi-organ failure from overwhelming sepsis following step up to diverticulectomy on day 7 (case 20). The mortality rate in primary operated patients was 6% (2/31), the causes of which were given as cardiac failure (case 32), and unknown (case 9).

Median hospital stay was 15.5 days after conservative management and 17 days after surgery. For surgically managed patients, three patients developed postoperative duodenal fistula. In two, duodenal fistula developed following simple diverticulectomy (cases 18 and 34). In another patient, a duodenal fistula developed following D3/4 duodenectomy (case 28). For conservatively managed cases with step up to surgery, one case (14) developed a duodenal fistula after Roux-en-Y gastric diversion.

## Discussion

Perforation is a rare, potentially life-threatening complication of duodenal diverticula. This systematic review summarizes the current evidence that patients with limited symptoms may safely and successfully undergo conservative treatment under close clinical observation. In patients with peritonitis or in those who fail conservative management, the data is less clear. Anatomical complexity and wide range of possible surgical manoeuvres highlights the need of an experienced and multidisciplinary team. Thorson’s series of 61 cases identified a trend towards CM, with 23% receiving CM [9]. The present series confirms this trend has been sustained with 34% undergoing initial conservative management, with or without step-up to SM.

### Diagnosis

The clinical picture in a patient with perforated duodenal diverticula is often heterogenous and non-specific. When symptoms do arise, the most common manifestations, including abdominal pain, vomiting and fever, are often non-specific, resembling those associated with the perforated peptic ulcers [25]. Accurate early diagnosis is crucial to facilitating conservative therapy or limited surgery. Clinical examination, vital signs, blood chemistry and radiological diagnostics all play an important role in the work-up and treatment of perforated duodenal diverticula. Specific CRP values were only given in 15 of the cases represented in this series. The CRP value was below 100 mg/L in 13/15 cases at presentation. Whilst the available data was insufficient to enable a statistically significant analysis, this observation may reflect the more limited inflammatory response which takes place in the retroperitoneal space. CT is the gold standard modality for the diagnosis of the perforated duodenal diverticulum. Key radiological findings are threefold: duodenal wall thickening ≥ 4 mm, mesenteric fat stranding and extraluminal or retroperitoneal air/fluid. The radiological entity known as diverticular microperforation describes a contained duodenal diverticular perforation with miniscule extraluminal air bubbles without evidence of abscess. From a radiological standpoint, a diverticular microperforation is particularly amenable to conservative treatment [26]. Analysis of the cases presented here demonstrates that CT was used as the primary diagnostic in 92% of the cases. This lies in stark contrast to 11% of cases published in previous series and represents an increasing trend towards routine implementation of CT imaging in the work-up of abdominal pain in the emergency department [9].

### Management: towards a systematic step-up approach

Non-operative management of a perforated duodenal diverticulum was first reported by Shackleton et al. in 1963 [27]. From this point until 1989, only five additional cases demonstrating conservative therapy were published. Thorson’s 2012 series of 61 cases first identified a trend towards conservative management, with 23% (14/61) of the patients having received non-surgical intervention [9]. The present series confirms this trend has sustained with 34% of patients successfully treated conservatively. Indeed, our own recent experience managing three cases of perforated duodenal diverticula in relatively young patients without signs of peritonitis proved successful using conservative therapy. In the present series, conservative treatment was reserved particularly for stable patients without signs of peritonitis. The components of non-surgical treatment were similar across cases and are summarized in Fig. 4. Percutaneous drainage was pursued as adjunct treatment in some cases. As previously mentioned, endoscopic insertion of a feeding tube post-Treitz combined with gastric lumen decompression was successfully used for the conservative management.

The criteria for operative management as derived from the present series included stable patients with failure to improve, patients with peritonitis and unstable patients**.** As portrayed in Table 2, in all 5 cases of failed conservative therapy, either clinical deterioration (sepsis, acute abdomen, hemodynamic instability) or persistent abdominal pain triggered the decision for repeat diagnostic imaging (CT) which, in turn, revealed a previously unseen small bowel perforation or worsening of a collection which, thus clinching the decision to step-up to surgical intervention. These findings underscore the importance of close clinical monitoring of patient’s with suspected duodenal diverticula perforation who are initiated on conservative management.

Once the decision for operative management was taken, intraoperative assessment was required to determine the most appropriate surgical technique. The intraoperative assessment can be organized into six main components summarized in Fig. 4. Of these six components, the three most important considerations are the extent of tissue friability, location of the diverticulum and size of the diverticular collar. In cases with limited tissue friability and small collar size, a simple diverticulectomy was shown to be a suitable technique for perforated diverticula located in the first, third, fourth portions of the duodenum, as well as diverticula in the second part of the duodenum which can be removed safely, without compromising the ampulla.

If the tissues have become friable, if the diverticular collar is extremely wide or if a large portion of the duodenal circumference is involved, single or double wall closure may become challenging, in which case more extensive surgery may be required. Again, the location of the diverticulum must be considered. In the context of D3/D4 diverticula, these are by virtue of anatomy amenable to a partial duodenectomy with end-to-end or end-to-side duodenojejunostomy. In the case of D2 diverticula, the surgical approach becomes more complex, ranging from gastric diversion procedures to duodenopancreatectomy (Whipple) if the periampullary region cannot be spared [23, 24]. Examples of gastric diversion techniques include pyloric exclusion with Roux-en-Y reconstruction [22], Billroth II or duodenostomy. As noted by Fujisaka, pyloric exclusion may be sufficient and in difficult cases closure of the perforation may not always be necessary. These are high-risk procedures; should the patient become unstable, the surgeon may be left with no choice other than placing a retro-peritoneal drain and aborting surgery. Unfortunately, specific information pertaining to diverticular collar size and circumference was not obtainable for most cases in the present series.

In terms of more invasive surgery, one case of pyloric exclusion with a Roux-en-Y reconstruction was reported for a perforated D2 diverticulum with extremely friable tissue. Additionally, two cases of Whipple procedure were recorded. The data from the present series suggests that whilst such complex procedures are required in some cases, the majority of perforated duodenal diverticula can be managed with less invasive surgery. Consideration of surgical complications such as bile duct injury, duodenal fistula or abscess also plays a crucial role in choosing the most appropriate approach. For example, according to Fujisaki et al., the high rate of fistula associated with a Billroth II procedure is a compelling argument for pursuing a Roux-en-Y reconstruction [22]. Biliary leaks should be managed by adequate external drainage and/or ERCP, papillotomy and biliary stent placement.

This study has some limitations: The first one is missing data parameters owing to heterogeneity in reported attributes across reports. Therefore, the comparability of patient’s baseline characteristics at the beginning of the treatment could not be fully assessed and so the results should be interpreted with caution. For example, additional risk factors which may orient treatment strategy towards conservative or surgical therapy were only reported in a proportion of the cohort, such as duration of symptoms (38% cases), vital signs (20%), temperature (46%), white cell count (68%) and CRP (49%). Additionally, the study has a relatively small sample size limiting the conclusions from the presented data.

## Conclusion

Optimal management of the perforated duodenal diverticula continues to be a challenging topic. Taken together, the present study observed a continued trend towards conservative management of this entity. We hypothesize that a key contributing factor may be the routine incorporation of computed tomography in the diagnostic work-up of patients presenting to the emerging room. Expertise with all the techniques summarized in this review, combined with careful clinical observation, is however crucial to allow safe implementation of conservative treatment with a step up to percutaneous drainage or surgery when necessary. In this manuscript, we leverage some of the observations collected during the review by offering a clinical algorithm (Fig. 4) which surgeons may find useful to help classify and stratify optimal treatment for these patients.
